# Understanding occipital pressure sores in UK military casualties: a pilot study in healthy military personnel

**DOI:** 10.1136/military-2022-002305

**Published:** 2023-02-01

**Authors:** Panagiotis Chatzistergos, T E Scott, M Thorburn, N Chockalingam

**Affiliations:** 1 Centre for Biomechanics and Rehabilitation Technologies, Staffordshire University, Stoke-on-Trent, UK; 2 Intensive Care Unit, University Hospital of North Staffordshire NHS Trust, Stoke-on-Trent, UK; 3 Academic Department of Military Anaesthesia and Critical Care, Royal Centre for Defence Medicine, Birmingham, UK; 4 Academic Department of Military Nursing, Royal Centre for Defence Medicine, Birmingham, UK; 5 Faculty of Health Sciences, University of Malta, Msida, Malta

**Keywords:** adult intensive & critical care, wound management, preventive medicine

## Abstract

**Introduction:**

The high prevalence of occipital ulcers in UK military casualties observed during the conflict in Afghanistan is a multifactorial phenomenon. However, the consensus is that ulceration is triggered by excessive pressure that is maintained for too long during the use of the general service military stretcher. Thresholds for capillary occlusion are accepted benchmarks to define excessive pressure, but similar thresholds for safe/excessive duration of pressure application do not exist. To address this gap in knowledge, we propose to use the time it takes for a healthy person to feel pain at the back of the head as an initial indication of safe exposure to pressure.

**Methods:**

Healthy military personnel (16 male/10 female) were asked to lie motionless on a typical general service stretcher until they felt pain. Time-to-pain and the location of pain were recorded. To support the interpretation of results, baseline sensitivity to pain and pressure distribution at the back of the head were also measured. Independent samples t-test was used to assess differences between genders.

**Results:**

Twenty participants felt pressure-induced soft-tissue pain at the back of the head. The remaining six participants terminated the test due to musculoskeletal pain caused by poor ergonomic positioning. On average, pain at the occiput developed after 31 min (±14 min). Female participants were significantly more sensitive to pain (t(24)=3.038,p=0.006), but time-to-pain did not differ significantly between genders (p>0.05).

**Conclusions:**

When people lie motionless on a typical military stretcher, the back of the head is the first area of the body that becomes painful due to pressure. The fact that pain develops in ≈30 min can help healthcare providers decide how frequently to reposition their patients who are unable to do this on their own. More research is still needed to directly link time-to-pain with time-to-injury.

**WHAT IS ALREADY KNOWN ON THIS TOPIC**

- The use of the typical military stretcher significantly increases the risk for occipital ulceration in military casualties.
- Ulceration is caused by exposure to high interface pressures at the occiput that are sustained for too long.

**WHAT THIS STUDY ADDS**

- The present study offers the first assessment of a threshold separating safe from excessive duration of pressure application.
- This indirect assessment is based on the time it takes for healthy people to start feeling pain at the occiput when lying motionless on the stretcher (time-to-pain).

**HOW THIS STUDY MIGHT AFFECT RESEARCH, PRACTICE OR POLICY**

- Time-to-pain measurements can help healthcare providers decide how frequently to reposition their patients on the stretcher to reduce the risk of ulceration.

## Introduction

**Figure 1:**
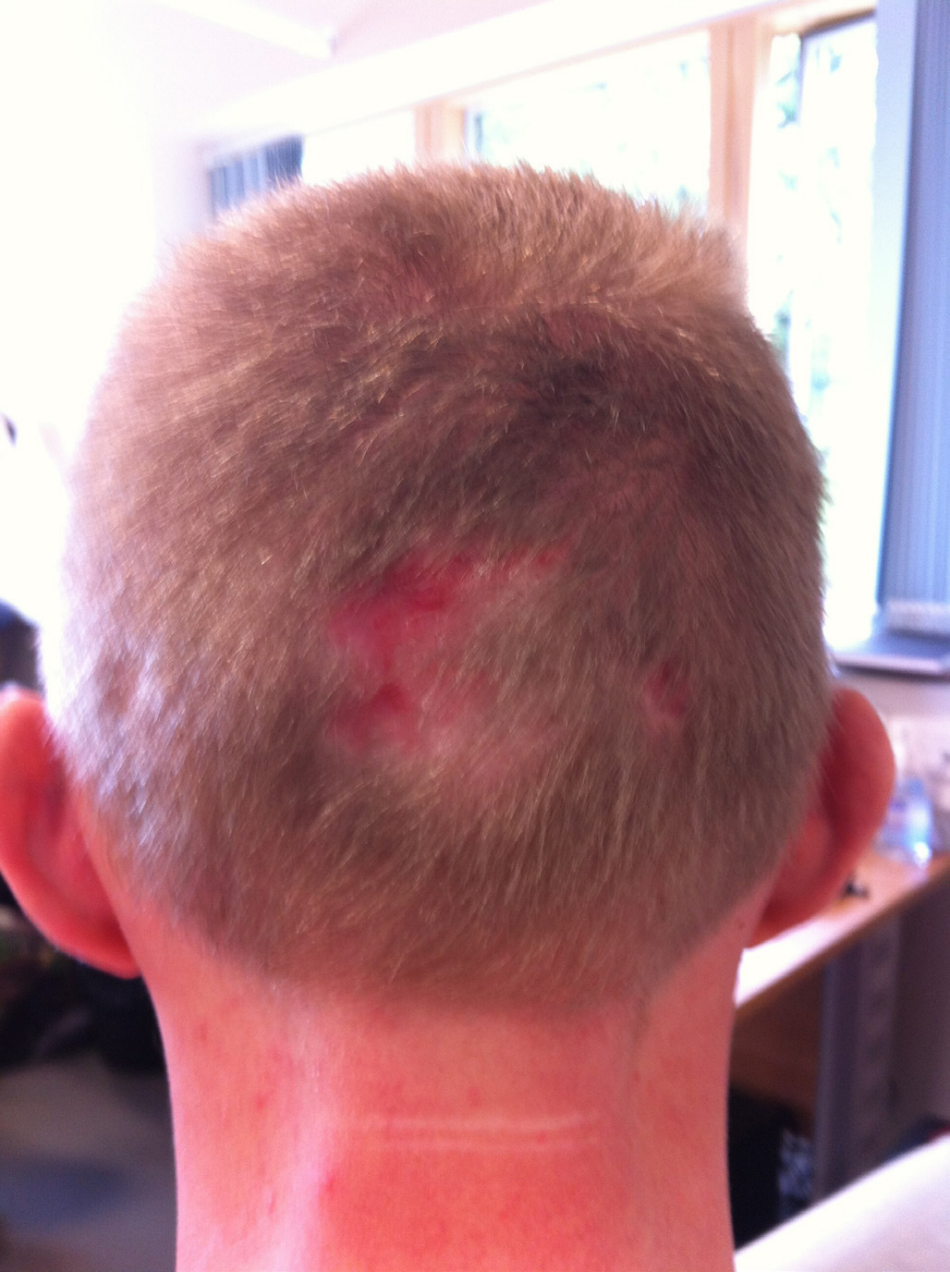
Example of an occipital pressure sore. Reproduced with permission from Scott *et al*.^3^

 Occipital pressure sores are immobility-related areas of tissue damage and hair loss that are developed at the back of the head. In historical civilian trauma cohorts, such ulcers were seen in approximately 1% of casualties.^1^ Occipital pressure sores in such circumstances were associated with the use of rigid cervical collars and pre-hospital spinal immobilisation.^2^ In UK military casualties during the conflict in Afghanistan, occipital pressure sores have only been identified in survivors requiring intensive care admission. These patients would have been stretcher cases on transfer to the hospital.^3^ In this cohort of patients, occipital pressure sores were seen in 33% of casualties attending a specialist rehabilitation unit after discharge from the intensive care unit (figure 1). Similarly, 19% of American casualties arriving at an equivalent rehabilitation facility suffered this injury.^4^ In 25% of UK casualties with occipital ulceration scarring and alopecia were permanent.^3^ Such an injury represents avoidable harm to those in our care and further blunts the casualty’s self-esteem and confidence. Moreover, on occasion, these wounds have required surgical repair which creates an unnecessary surgical burden.^3 4^

The aetiology of pressure sores is not yet clearly defined. While it is likely to be multifactorial,^5^,^9^ the consensus is that tissue overloading is among the key contributors to the risk for ulceration.^6 10 11^ Previous research has indicated that the pressure developed at the back of the head when lying on a typical general service stretcher is significantly higher compared to the stretchers used in civilian practice.^12 13^ The observed pressures quickly cause capillary occlusion and, if occlusion is sustained, lead to ischaemia and even to tissue necrosis.^12^,^15^ Moreover, the viscoelastic nature of soft tissues means that prolonged exposure to pressure could also lead to substantial compressive deformations in the tissue causing an injury that would not have happened if the same pressure was sustained for less time. Therefore, both the duration and the magnitude of pressure are equally important in the aetiology of injury and need to be considered when exploring the pathways to tissue overloading.^7 16^

Understanding what constitutes safe exposure to pressure and what is potentially injurious is very important for the design of effective mitigation strategies against pressure ulcers. Retrospective studies involving patients who developed pressure ulcers after surgery have indicated that, depending on the intensity of loading, pressure ulcers can develop even within an hour of continuous exposure to pressure.^17^ However, information on the timeframe of ulceration remain limited.^16^

To address this major knowledge gap, this study proposes an indirect assessment of the duration of safe exposure to pressure. Healthy people already have a sophisticated mechanism to detect potentially injurious exposure to pressure, through the sensation of pain.^18^ When exposure to pressure becomes excessive, pain ‘motivates’ us to offload and redistribute pressure to protect the affected tissues. Based on that, we hypothesise that the time it takes for a healthy person to feel pain at the back of the head when lying still on the stretcher offers an initial indication of how long it is safe to maintain pressure. Our study presents the first normative data on time-to-pain complemented by measurements of sensitivity to pain and pressure distribution.

## Participants and methods

### Participants

Healthy military personnel aged between 18 and 50 who were fit to deploy were included in this study. People with a medical history of pressure ulceration, pregnant women or nursing mothers were excluded. Volunteers using pain-killing medication in the previous 24 hours leading to the experiment, psychoactive medication or medication that can cause vasodilation at the occiput were also excluded. Following screening against the inclusion-exclusion criteria, 26 (16 male/10 female) people were included in total in this study (table 1). Key anthropometric parameters (stature, body mass, shoulder breadth) were also measured to ensure that the group of recruited participants was representative of the UK^19^ and NATO military populations.^20^

**Table 1:** Demographic/anthropometric data,^19 20^ measurements of pain pressure thresholds (PPT), peak pressure at the back of the head, time-to-pain and the self-assessed severity of the experienced pain (0=no pain, 10=worst pain possible)^21^. Average (±SD) results are presented separately for the entire tested population, male and female participants.

	Total	Male	Female	P value†
Number	26	16	10	–
Age (years)	33.5 (±8.3)	33.0 (±8.3)	34.4 (±5.5)	0.644
Body mass (kg)	81 (±11)	85 (±9)	76 (±12)	0.054
Stature (m)	1.760 (±0.085)	1.799 (±0.070)	1.697 (±0.068)	0.001‡
Stature normalised over stretcher length*	0.922 (±0.044)	0.942 (±0.037)	0.889 (±0.036)	0.001‡
Body mass index (kg/m^2^)	26.4 (±3.1)	26.2 (±2.2)	26.5 (±4.2)	0.839
Shoulder breadth (m)	0.496 (±0.045)	0.511 (±0.043)	0.471 (±0.067)	0.022‡
PPT forehead (kPa)	195 (±88)	224 (±85)	158 (±80)	0.061
PPT arm (kPa)*	171 (±67)	204 (±65)	130 (±54)	0.006‡
Pressure at occiput (kPa)	59 (±17)	60 (±16)	57 (±19)	0.683
Time-to-pain (min)	32 (±14)	32 (±13)	29 (±17)	0.630
Pain scale (0–10)	2.7 (±1.1)	2.5 (±1.1)	2.9 (±1.0)	0.398

* The relative participant stature to stretcher dimensions is presented by dividing stature over stretcher canvas length (ie, 1.91m).Average (Standard deviation) results are presented separately for the entire tested population, male and female participants.The values of the independent samples t-tests that were used to assess differences between genders are also shown. Statistically significant differences between genders are indicated with an asterisk.

† The p values of the independent samples t-tests that were used to assess differences between genders.

‡ Statistically significant difference between genders (ie, P<0.05).

### Measurements

The main outcome measure of testing was the time it took to start feeling pain at the back of the head (ie, time-to-pain). The participants were asked to lie motionless on a general service stretcher. More specifically, they lied in a supine position until they started feeling pain in the occipital area of their heads or any other part of their body. It was emphasised to them that this was not a test of endurance and that they should sit up and terminate the test when they first felt pain, not when pain became unbearable. They were also informed that the test would be terminated by the researcher after 60 min. If the participant terminated the test because of pain or discomfort, they were asked to indicate the exact area where pain/discomfort was felt. If the test was terminated due to pain/discomfort at the occiput, they were also asked to score the pain experienced by drawing a line on a standardised 0–10 numeric pain rating scale (0=no pain, 10=worst pain possible).^21^ Testing took place indoors (temperature 20°C (±2°C), humidity 48% (±12%)) in a quiet room and special attention was paid to deprive the participants of any information about the time during testing.

**Figure 2:**
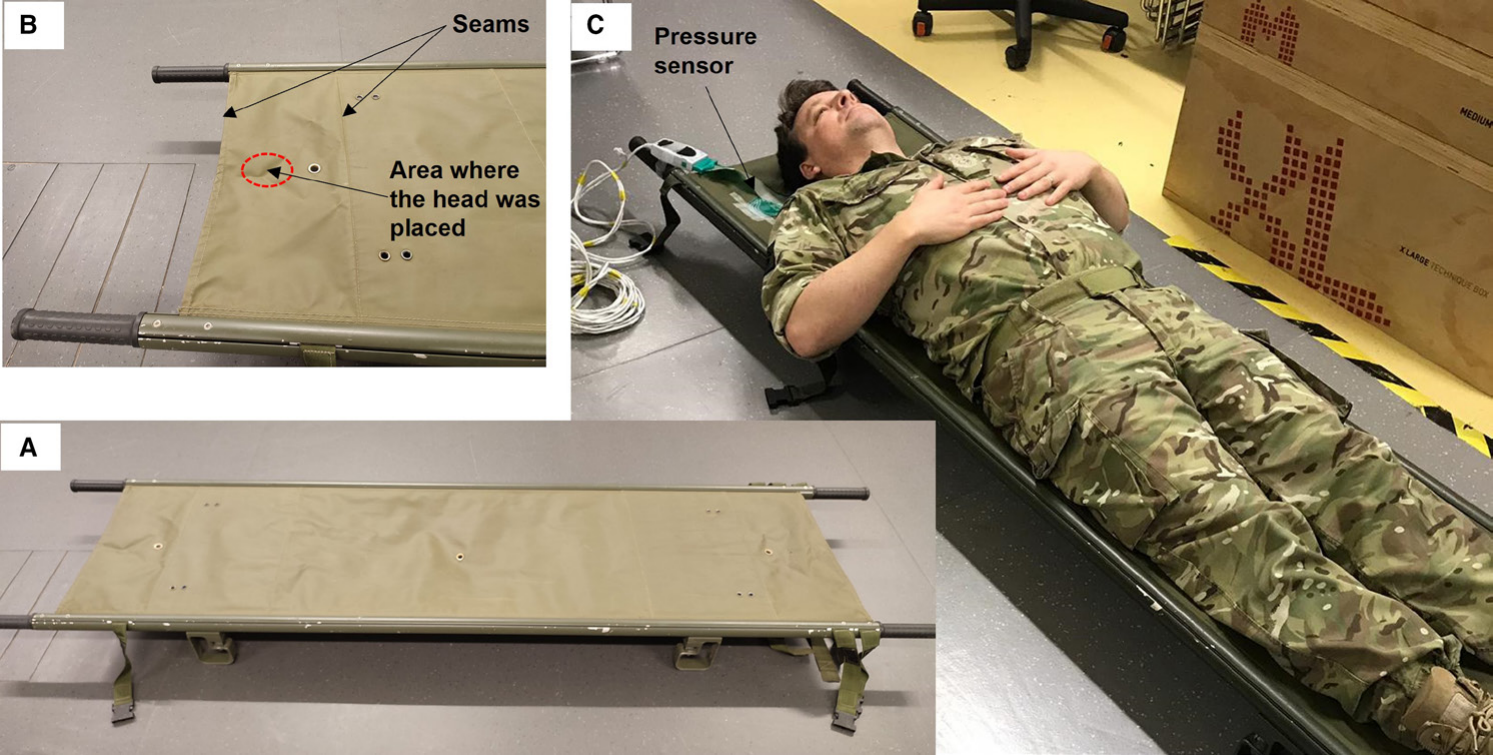
The military stretcher that was used in this study (**A**) and a closer look at the head rest area (**B**). The testing set up for the measurement of pressure distribution is also shown (**C**).

All tests were conducted on the same stretcher (figure 2A). This type of stretcher is used across all three services of the UK Armed Forces and follows a design which is replicated across NATO countries.^12^ The stretcher canvas material was 1.91 m long and 0.49 m wide. When the participants were positioned on the stretcher, they were asked to avoid the seams of the stretcher material and other features on the material surface that might lead to increased pressure at the back of the head (figure 2B). Even though the stretcher length was greater than the stature of almost all participants (table 1), correct positioning of the head, in some cases, meant that their legs were hanging out of the stretcher canvas.

Time-to-pain can be affected by an individual’s sensitivity to pain as well as by the magnitude of pressure that is developed at the occiput when lying on the stretcher. To support the interpretation of time-to-pain results, baseline sensitivity to pain and pressure distribution were also measured at the beginning of each testing session.

Measurements of sensitivity to pain were performed with the help of a standardised algometer (Citec) using an established protocol.^22^ The algometer is in essence a handheld dynamometer with a cylindrical punch (footprint 1 cm^2^) attached to its loading end. The cylindrical punch was slowly pressed against the skin surface with increasing intensity until the participant verbally indicated the start of mild pain. This procedure was first demonstrated and then repeated three times. The pain pressure threshold (PPT) was assessed as the average of three measurements of maximum force. To ensure the measurement of PPT is relevant to the specific application but at the same time it does not affect the area of interest (ie, the back of the head), sensitivity to pain was assessed at the forehead. The forehead is one of the main sites in the head to assess pain, but this measurement has been found to be prone to high intra-individual variation.^23^ To account for this, PPT was also measured in a muscle of the lower arm where higher reliability can be achieved.^23^

Occipital pressure distribution was measured using a thin pressure sensor (thickness 0.152 mm) with a spatial resolution of 0.5 cm^2^ (F-Scan; Tekscan, USA). The sensor was placed between the participant’s head and the canvas of the stretcher and peak pressure was recorded for 10 s (figure 2C). The sensor was calibrated according to the manufacturer’s instructions before each testing session and was completely removed from the stretcher for the time-to-pain measurement. Three recordings were performed and the average peak pressure was recorded. From this point on, ‘average peak pressure’ will be referred to simply as ‘peak pressure’.

Because of the potential effect hair might have on pressure distribution, the length of freely hanging hair at the back of the head was also measured (occipital scalp to end of longest hair) prior to lying on the stretcher. Based on that, participants were divided into three subgroups: (A) short hair (length ≤1 cm), (B) medium hair (1 cm < length ≤ 5 cm) and (C) long hair (length >5 cm).

### Statistical analysis

The normal distribution of results was confirmed using the Shapiro-Wilk test. Pearson correlation analysis was used to investigate the association between time-to-pain, demographic, pain sensitivity and pressure measurements. Linear regression analysis was used to assess how much of the variability in time-to-pain can be explained by variability in PPT and peak pressure. Separate regression analyses were conducted for forearm and forehead PPT. Independent samples t-test (equal variance assumed) was used to assess differences between the results for male and female participants and between people with short, medium and long hair. All statistical analyses were conducted using IBM SPSS Statistics V.26.

## Results

**Figure 3:**
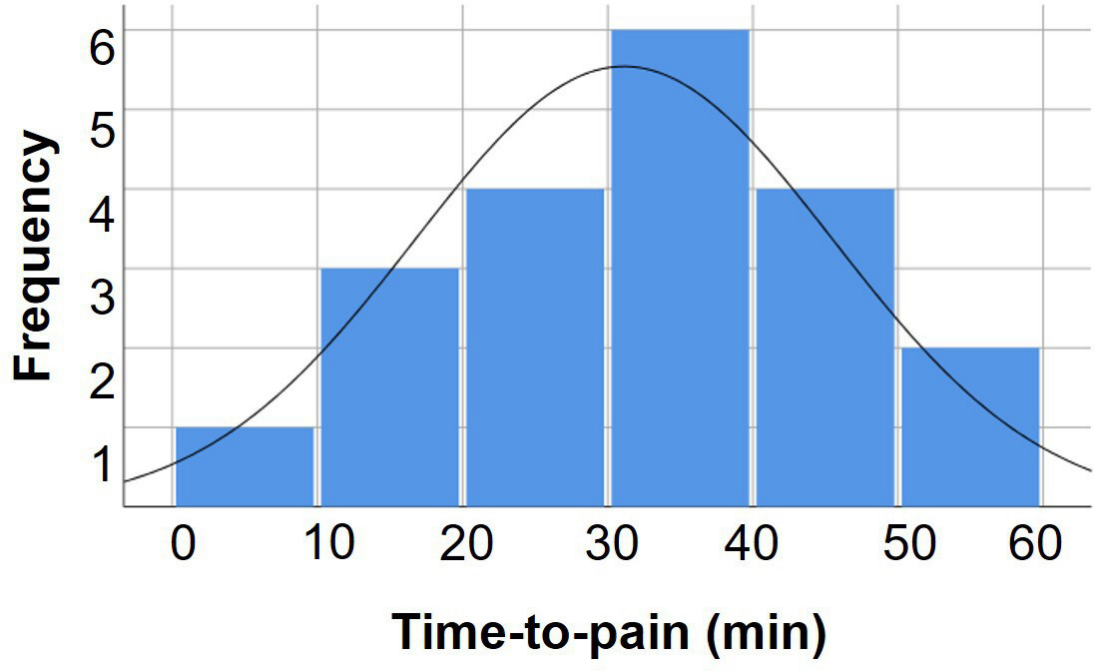
Histogram of the distribution of time-to-pain measurements. The assumed normal distribution is also shown.

Out of the 26 participants, 20 (12 male/8 female) terminated the time-to-pain test due to pain/discomfort at the occiput. On average, these participants felt pain at the occiput after 31 min (±14 min) and rated this pain as 2.7 (±1.0) out of ten.^21^ The distribution of time-to-pain measurements can be seen in figure 3.

From the remaining six participants, three terminated the test because of pain in the neck, two because of low back pain and one because of leg pain. All participants terminated the measurement of time-to-pain within the predefined 60 min window.

Pearson’s correlation analysis did not reveal any statistically significant association between time-to-pain and any of the demographic, PPT or pressure measurements. Comparison between the results for men and women revealed significantly lower PPT at the forearm in women (95% CI 2.39 to 12.49, t(24) = 3.038, p=0.006). As expected, women also had smaller stature (95% CI 4.39 to 15.98, t(24) = 3.630, p=0.001) and smaller shoulder breadth (95% CI 0.63 to 7.42, t(24) = 2.450, p=0.022) than men. No other statistically significant difference was found between genders (table 1). Linear regression analysis indicated that time-to-pain cannot be statistically significantly predicted based on peak pressure and PPT (at the forearm or forehead).

**Figure 4:**
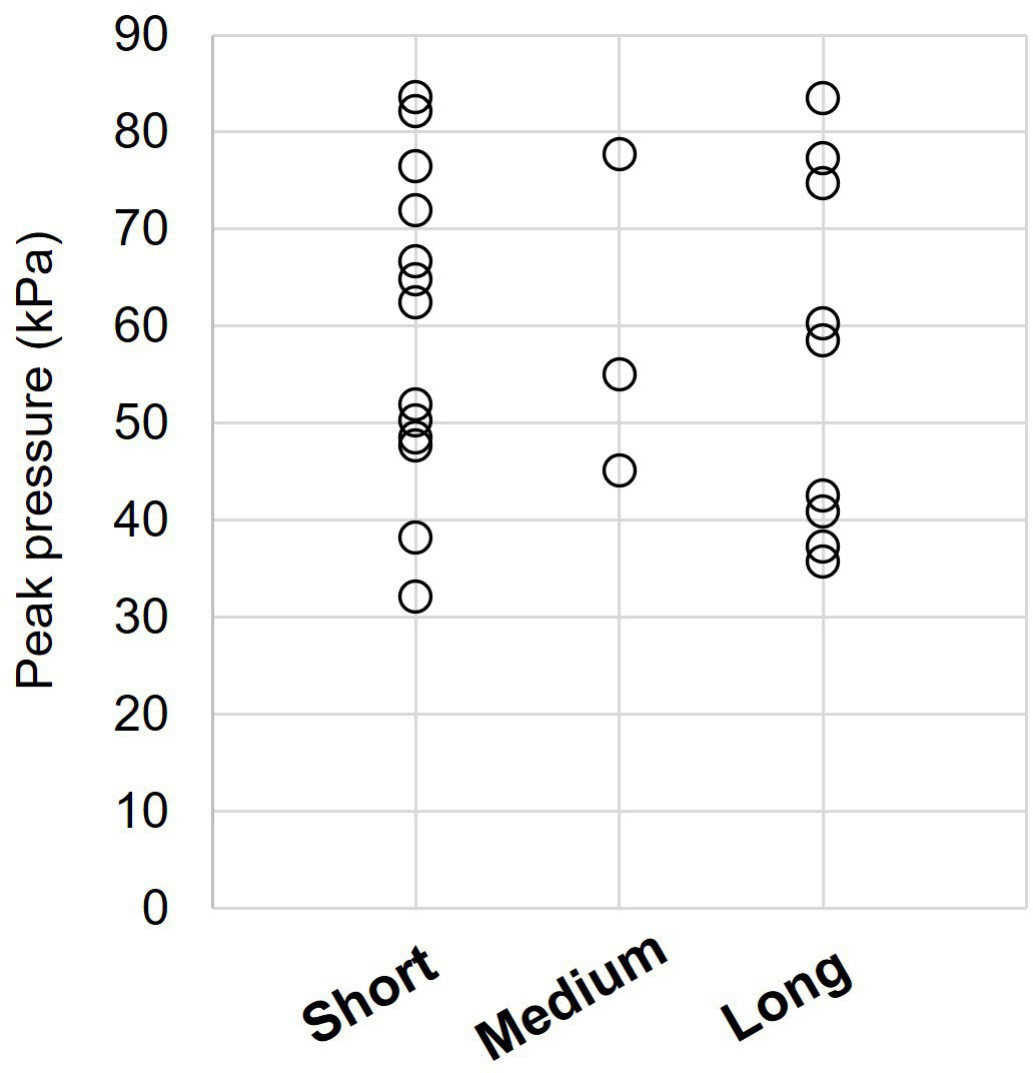
Distribution of peak pressure measurements for participants with short hair (hair length ≤1 cm), medium length hair (1 cm < hair length ≤ 5 cm) and long hair (hair length >5 cm).

With regards to hair length, thirteen people had short, three had medium and nine had long hair. The length of hair did not appear to have a significant effect on pressure (figure 4). Indeed, the average pressure in people with short hair (n=13) or in people with long hair (n=9) was 59 kPa (±16 kPa) and 56 kPa (±19 kPa), respectively. Independent samples t-test indicated that this difference in pressure was not statistically significant (95% CI −12.64 to 18.63, t(20) = 0.40, p=0.69).

## Discussion

The development of pressure ulcers is multifactorial.^5^,^9^ At the same time, the consensus is that ulceration is triggered by prolonged exposure to pressure.^6 12 13^ An assessment of the thresholds of exposure to pressure that separate safe loading from overloading would significantly advance our understanding of the aetiology of ulceration and lead to better prevention.

As it stands, the direct measurement of overload thresholds in the context of occipital ulceration would involve applying and sustaining pressure until the development of an overload injury in the soft tissues at the back of the head. Similar methodologies have been successfully used in literature to study overload injury in the muscle of animal models of pressure ulceration^24^,^26^ or in in vitro tissue engineering models.^27^ However, this invasive approach is not applicable for human in vivo testing.

To overcome this impasse, we are proposing a new indirect method of studying in vivo overloading in humans. We propose that excessive exposure to loading is not defined as the loading that causes injury, but as the clinically relevant loading that causes pain in a tissue with normative sensitivity to pain.^18^

Out of 26 participants in total, 20 terminated the test because of pain at the occiput and 6 (23%) because of pain in other parts of their bodies. However, none of these six people felt soft-tissue pain that was caused by excessive exposure to pressure that could be linked to the risk of developing a pressure ulcer somewhere other than the occiput. Their pain was musculoskeletal in nature resulting from poor ergonomic positioning of their neck, back or legs.

The fact that the back of the head was the first area where pressure-induced soft-tissue pain was felt aligns with existing data that highlight the occiput as the most frequent area affected by ulceration.^4^ A retrospective audit of patient data of US military casualties from Operation IRAQI FREEDOM who were admitted to a polytrauma rehabilitation centre showed that occipital ulcers accounted for 50% of all pressure lesions and were also more severe than those of the sacrum or extremities (100% of the stage III, IV lesions, and 72% of the scars or eschars were at the occiput).^4^

Moreover, measurements of interface pressure from the literature have indicated that when a person is lying supine on a general service military stretcher, the highest pressures are developed at the back of the head and that their magnitude is significantly higher than thresholds for capillary occlusion.^12^ Although the actual pressure values are not comparable between studies due to the nature of the technologies used, the latter finding was also confirmed by the present study. More specifically, the average pressure in the population tested here was 59 kPa which is almost 15 times higher than the reported threshold for skin capillary occlusion of 4 kPa (30 mmHg).^15^ Having longer hair also did not appear to offer significant protection against high pressures (figure 4).

With regards to the key outcome measure of this study, namely time-to-pain, the results presented here suggest that, on average, healthy people started feeling pain at the back of their heads after approximately 30 min of lying still on the stretcher. Combined with relevant literature on the timeframe for ulceration, this finding can inform further research on time-to-pain and lead to effective mitigation strategies for the risk of occipital ulceration.^16 17^

More specifically, a retrospective study of pressure ulcers in people undergoing surgeries of known duration indicated that, interface pressures exceeding the systolic pressure led to the development of muscle trauma and pressure ulcers within 6 hours of continuous exposure to loading. However, the injurious duration of exposure dropped to less than an hour when the magnitude of pressure increased to (approximately) four times the systolic pressure.^16 17^ Based on this and considering the magnitude of the observed pressures at the occiput (ie, 15 times higher than the capillary occlusion threshold), it can be hypothesised that occipital ulceration develops in less than an hour of continuous exposure to pressure. Our finding that a person with normative sensitivity to pain would feel pain at the occiput in about half an hour substantiates this hypothesis.

In the absence of a modified or redesigned military stretcher that significantly reduces the magnitude of pressures on the occiput, controlling the duration of sustained pressure remains one of the key mitigation strategies for the prevention of occipital ulceration.^28^ However, as it stands, there are no clear guidelines on the frequency of repositioning for effective ulcer prevention. Even though we cannot conclude at this stage how long it will take for an overload injury to develop at the back of the head, this study showed that people with normative sensitivity to pain (on average) would not allow pressure to be sustained for longer than half an hour. This information can be useful to deployed healthcare providers supporting them to make informed decisions on how frequently to reposition the heads of patients who are unable to do this on their own. Evidence-based guidelines that reduce the risk for occipital ulceration are particularly relevant for the effective and safe use of the general service stretcher as a hospital bed or during the air transfer of casualties.^29^ This is especially relevant to casualty care in the ‘prolonged hold’ scenario associated with small-scale contingency operations.

The key limitation of this study is that time-to-pain was not directly linked to the risk of overload injury or ulceration. As a result, it is not possible to directly translate the measured time-to-pain to an assessment of time-to-injury. A direct causal pathway between exposure to pressure that causes mild pain and a physiological or biomechanical measurement directly linked to soft-tissue injury will be needed to establish the clinical relevance of time-to-pain. Another limitation of measurements of time-to-pain to study overloading is that, in their current form, they are applicable only for clinically relevant loading and only in tissues with normative sensitivity to pain. In other words, conditions where the body’s inherent ability to sense potentially injurious loading is not confounded by extrinsic (eg, method of loading application, environmental conditions) or intrinsic factors (eg, medication, distracting injuries). As a result, measurements of time-to-pain are limited to healthy individuals. Environmental factors like temperature and humidity are known to affect sensitivity to injury.^30 31^ For this study, temperature and humidity was controlled. Further research will be needed to explore their effect on time-to-pain.

## Conclusions

This study confirms previous findings in the literature indicating that the pressure developed at the back of the head while lying on a typical military stretcher is substantially higher than thresholds for occipital capillary occlusion. More research is still needed to directly link time-to-pain with time-to-injury. At the same time, appreciating that a healthy person with normative sensitivity to pain would choose to reposition themselves after ≈30 min can contribute to the development of evidence-based guidelines for the safer use of military stretchers.

## Data Availability

Data are available on reasonable request.
